# Estimation of body size and growth patterns in Korean boys

**DOI:** 10.1186/s40101-015-0058-2

**Published:** 2015-04-24

**Authors:** Youngsuk Lee

**Affiliations:** Department of Clothing & Science, Chonnam National University, 300 Yongbongdong, Gwangju, 500-757 Korea

**Keywords:** Anthropometry, Morphology, Proportion, Growth

## Abstract

**Background:**

Anthropometric surveys devised by each country attempt to fulfill the requirements of the manufacturers, designers, and human welfare device production, providing them with data and tools and allowing them to face both the internal and export markets. To this end, national anthropometric data collections and comparisons including three-dimensional information, together with comparison of these data among countries, are conducted at both the domestic and global levels.

**Methods:**

The anthropometric data of the Korean population measured in 2013 (Korean Agency for Technology and Standard (KATS) 2013 data) and the data collected from 2010 (KATS 2010 data) that was conducted on 710 males between the ages of 13 and 18 years were analyzed in this section to obtain information on Korean boy’s physical features and growth.

**Results:**

The mean height increased about 5 cm from 13 to 14 years which shows the early fast maturing somatotype. Also, the mean height of boys aged from 15 to 16 increased about 1 to 2 cm. For the results of body proportion rate index against height, they show 0.93, 0.81, 0.38, 0.99, and 0.26 times the height in eye height, shoulder height, fingertip height, and span and maximum shoulder breadth, respectively, in 16-year-old boys. For the body mass index, the weight is increased from the age of 16 years.

**Conclusions:**

There are several studies that cover growth features of the entire range from birth to maturity, and they have reported the comparison of the growth patterns among Europeans. Even though such researches have been made, as for the industry, the human modeling tools based on the anthropometric data and morphological features that cover all the countries should be developed for well-fit garments and other human-oriented design process.

## Background

Over the last hundred years, a great quantity of anthropometric data has been accumulated, although this was obtained neither for the sizing of clothes nor for artistic applications. Some data was obtained for purposes of taxonomy, seeking out similarities and differences among peoples; for physiology, in attempts to explain variation in body function; for clinical research to study abnormalities; and by anthropologists interested in the gradual development and evolution of man [1,2].

In recent years, anthropometric studies have mostly been directed toward the efficient operation of machinery and equipment and special attention being paid to the correct positioning of controls and to suitable seating arrangement [3,4]. From the early days of ergonomics, in fact, before the name was coined, it was recognized that individuals within a given population varied dimensionally, and that it was, therefore, necessary to design equipment with this in mind. Similarly, to indicate the men’s state of health and nutrition, and often indeed their physiological situation also, a child growth rate reflects, perhaps better than any other single index, the average of a nation’s public health and the average nutritional status of its citizens, when appropriate allowance is made for differences, if any, in genetic potential [5].

Thus, a well-designed growth study is a powerful tool which can monitor the health of a population, or pinpoint subgroups of a population whose share in economic and social benefits is less than it might be. There are certainly large differences between populations, in height and weight and age of puberty, for example, and it is now clear that a portion of these differences is genetic in origin and a portion (in the developing countries, a large portion), environmental [6]. Moreover, the accurate statistics of body features classified by country, age, and gender are also important factors, notably in multiracial societies. Indeed, such statistics allow defining the average virtual individual shape for each class to recognize the growing importance of some of them and to recognize the changes occurring with the passage of time.

Therefore, anthropometric surveys devised by each country attempt to fulfill the requirements of the manufacturers, designers, and human welfare device production, providing them with data and tools and allowing them to face both the internal and export markets [7]. To this end, anthropometric data collections and comparisons including three-dimensional information, together with comparison of these data among countries, are conducted at both the domestic and global levels [8,9].

In Korea, the first national anthropometry survey was conducted in 1979 by a Korean government division, the Korean Agency for Technology and Standard [10]. At that time, data were collected concerning 17,000 samples residing in various parts of the country aged between 6 and 50. A total number of 117 measurement dimensions were taken using calipers and tape measures.

Following this survey, the Korean government has been presenting a national anthropometric survey every 5 or 6 years. The surveys of 1986 [11], 1992 [12], 1997 [13], 2003 [14], and 2010 [15] were performed according to the following sequence: The survey was performed with the traditional measurement method (1D) using an anthropometer, somatometer, caliper, and tape measure. The 3D body scan data collection (using Body Line Scanner from Hamamatsu Co., Japan, Artec Body scanner, Russia) method (3D) was also adopted in order to obtain a good compromise and to modernize the fit and construction of their garments for the 2003 and from 2010 to 2013 surveys [16]. All body dimensions were measured with the method defined by the ISO 3635 [17], ISO 8559 [18], and ISO 20685 [19].

This paper deals with growth pattern of height, key dimension distributions according to height, and also morphological growth patterns of 6- to 20-year-old Korean boys using the Korean Agency for Technology and Standard (KATS) data taken from 2010 survey. It also investigates the comparison of the body mass index (BMI), body silhouette images, and the difference of mean heights between 2010 data and 2013 data of 13- to 18-year-old boys based on the anthropometric data samples of KATS taken from the 2013 survey.

## Methods

### Subjects

The anthropometric data of the 2,978 Korean males between the ages of 6 and 20 years measured in 2010 (KATS 2010 data) were analyzed in this paper to obtain the information on Korean boy’s physical features and growth patterns. The anthropometric data of Korean measured in 2013 (KATS 2013 data) which was consisted of 714 males between the ages of 13 and 18 were also used. The subjects were consisted of school boys selected at random from different urban regional zones.

Data on size and annual growth changes of 2013 measurements and 2010 KATS data samples were compared to clarify the tendency of time series data of maturation in body size. This study was approved as a research exempted from IRB oversight by the Institutional Review Board at the Chonnam National University(1040198-150427-HR-020-01).

### Anthropometry measurements

In this paper, the anthropometric data included stature, three girth dimensions (chest girth, waist girth, and hip girth), and arm length were selected to analyze the body size and growth tendency.

Five height dimensions (waist height, shoulder height, illiac spine height, crotch height, and hip height) and arm span were adopted to calculate for the values of the body proportions.

The one-dimensional survey was performed with the traditional measurement method (1D) using an anthropometer, sliding caliper, spreading caliper, large sliding caliper, and tape measure.

The 3D body scan data collection (using Body Line Scanner from Hamamatsu Co., Japan, Artec Body scanner, Russia) method (3D) was also adopted in order to obtain a body shape data.

Measurement performed with the basic standing posture, subjects stand erect with feet together, the shoulders are relaxed, and the arms are hanging down naturally.

### Anthropometry data analysis

The difference of mean values of heights between ages is used to compare the size distribution range and differentiate, to analyze the growth patterns of Korean boys according to ages.

The distributions of the size between height and chest and waist and hip girth values are used to distinguish the body size ranges in subjects and body type features.

The percentage of achieved growth rate (%) to adult size of height and girth dimensions in ages 6 to 20 years was indicated using the 2010 data.

The height comparison between 2010 and 2013 data in ages 13 to 18 years was also analyzed to presume the tendency of the maturation according to the time series data.

The 3D body shape modeling of the standard body shape of 13- to 18-year-old males obtained from 2013 KATS data is presented to define the Korean’s morphological features.

### Body proportions (calculated)

The proportion ratio index of five body height dimensions (waist height, shoulder height, illiac spine height, crotch height, and hip height) and arm span corresponding to total height dimensions are calculated to indicate child-adult transformation in body shape and body proportions.

### Body mass index

BMI was calculated as elements for considering the nutrition and healthy condition, using the World Health Organization (WHO) recommendations for BMI calculation: weight (in kg) divided by the height (in m^2^).

According to the international classification guidelines by World Health Organization, men with a BMI greater than 25 (kg/m^2^) were considered obese, and men with a BMI greater than 18.5 (kg/m^2^) but less than 23 (kg/m^2^) were considered in the normal range group. Men with a BMI greater than 23 (kg/m^2^) but less than 25 (kg/m^2^) were considered overweight, and men with a BMI less than 18.5 (kg/m^2^) were considered underweight.

## Results

### Size data and annual growth changes of measurements

**Figure 1:**
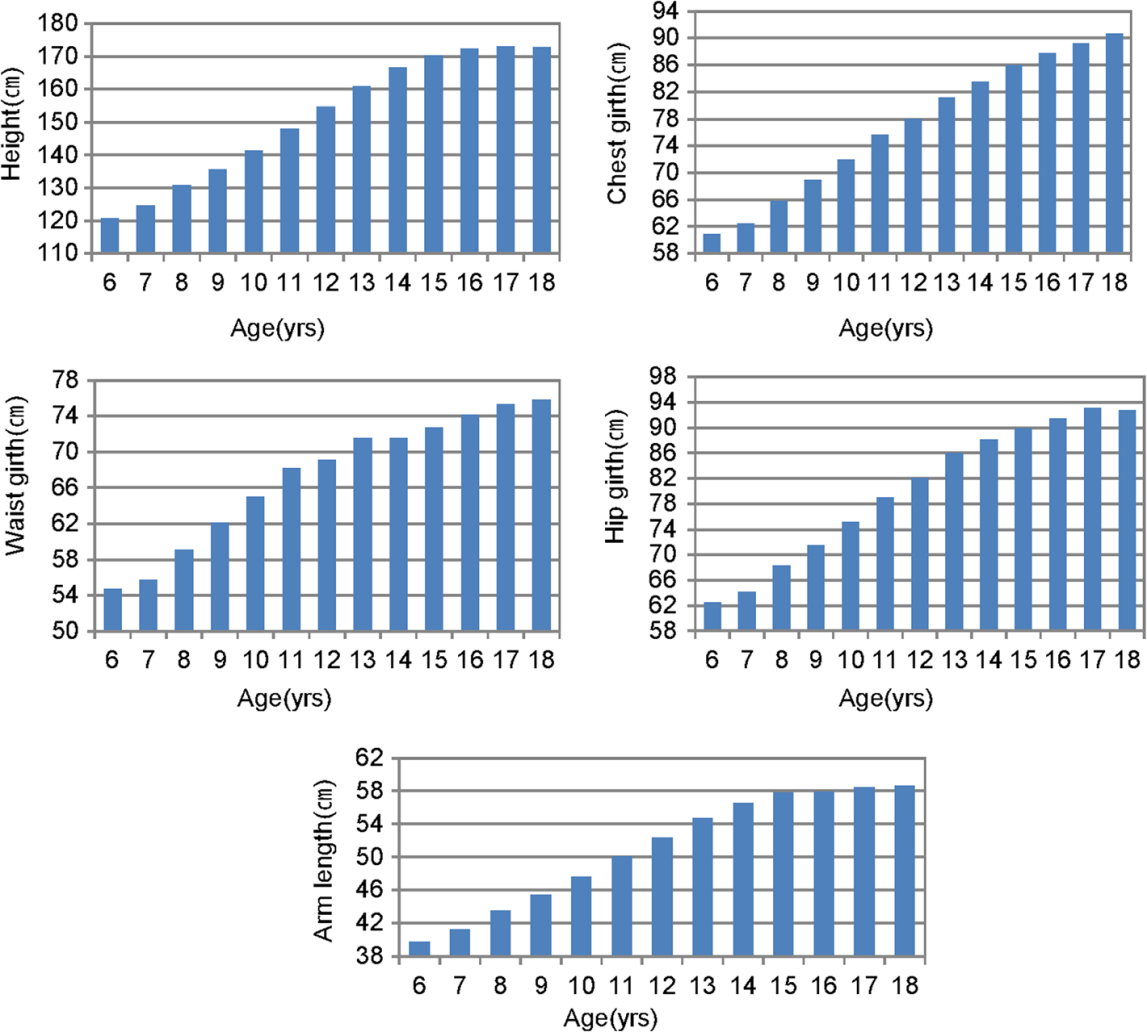
Size changes of measurements(2010 data).

The size changes of height, arm length, chest girth, waist girth, and hip girth measurements, which represent body development, and annual growth changes of measurements by ages 6 to 18 years in Korean boys are shown in Figure 1.

At age 6, the mean values of height, arm length, chest girth, waist girth, and hip girth were 120.7, 39.8, 60.9, 54.7, and 62.5 cm, respectively. Each year, height increases 4 to 7 cm between the ages of 6 and 14 years and 2 to 4 cm per year in other girth measurements (chest and hip). Arm length has increased about 1 to 2 cm per year between the ages of 6 and 16 years.

The ultimate size and shape that a child attains as an adult size are reached during 17 to 18 years of age.

### The distribution between height and girth measurements

**Figure 2:**
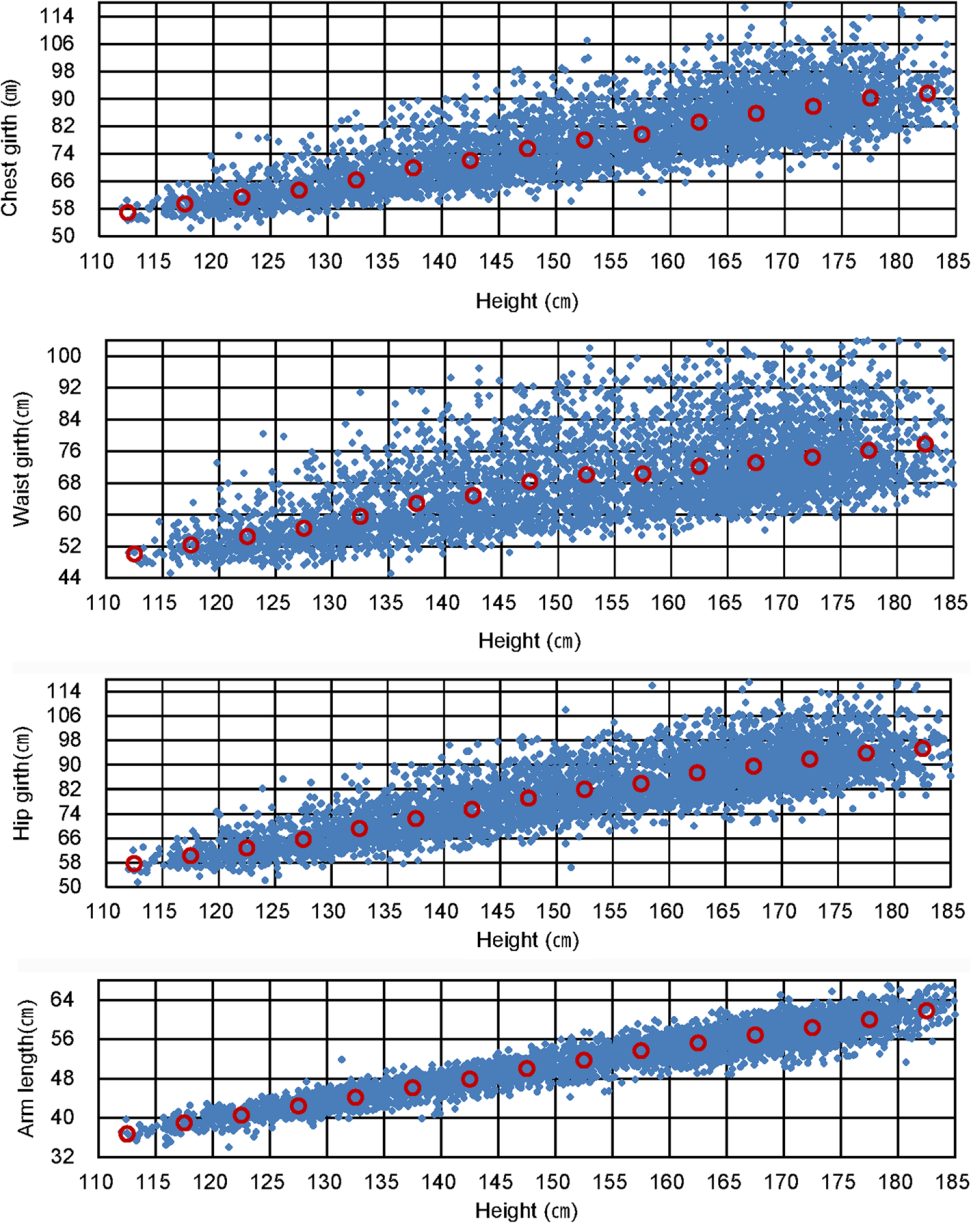
Size plotting of chest, waist, hio girth and arm length according to height.

Figure 2 suggests the correlations between height and chest girth, waist girth, and hip girth based on the data of body dimension taken in 2010 of subjects aged from 6 to 18 years. The values of chest girth are concentrated in the range from 75 to 94 cm with the height range of 160 to 170 cm which approximately represent 80% cover rate of 13- to 15-year-old males. The values of waist girth are concentrated in the range from 64 to 78 cm with the height range of 160 to 170 cm which approximately represent 60% cover rate of 13- to 15-year-old males. The values of hip girth are concentrated in the range from 78 to 94 cm with height range of 160 to 170 cm which approximately represent 70% cover rate of 13- to 15-year-old males.

Sixteen- to 18-year-old males who have a chest value between 95 and 105 cm belong to body size type L. This type indicates that he has a big chest compared to that of body size type M (chest value of 85 ~ 95 cm).

### Percentage of the achieved adult size in key measurements

**Figure 3:**
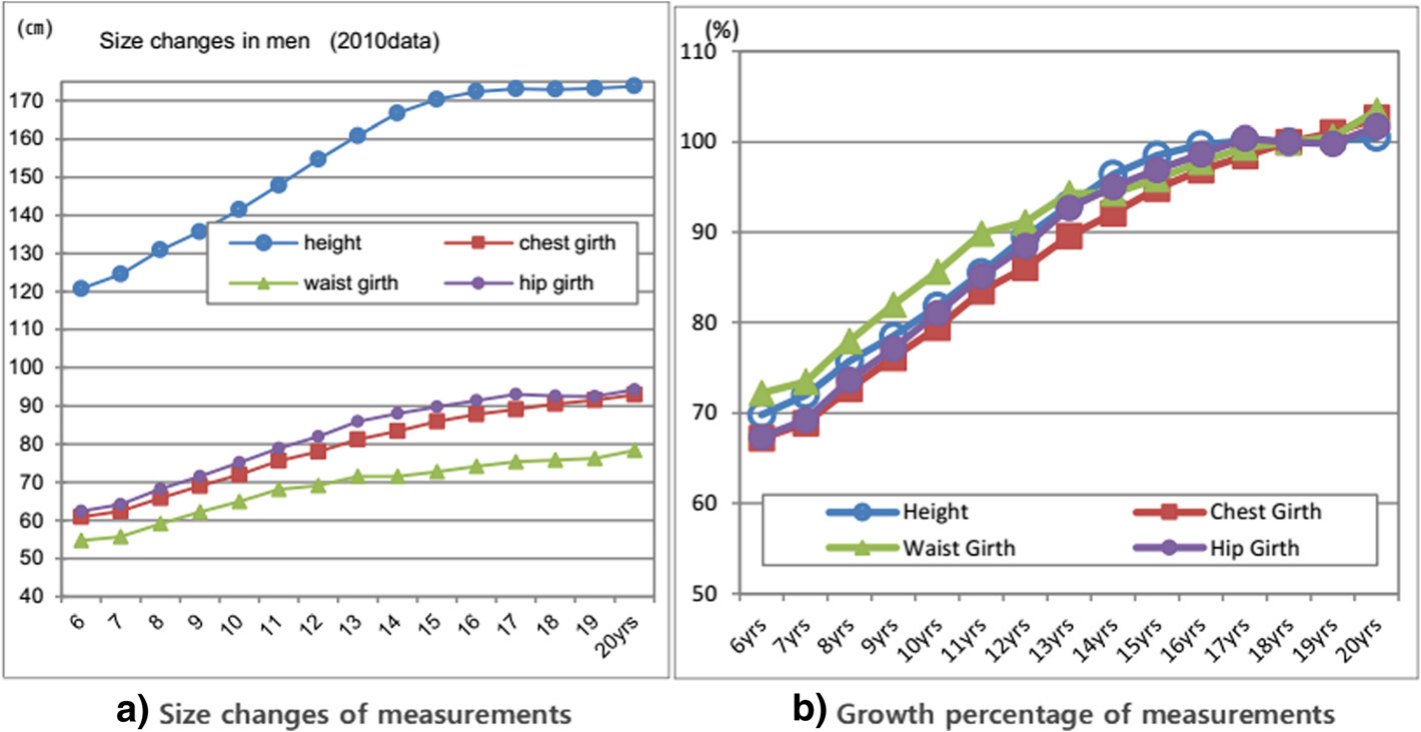
Size changes **(a)** and growth percentage **(b)** of measurements(2010 data).

The annual achieved size percentage to adult size of mean heights at ages 6 to 20 years and the growth pattern of other girth measurements at each age based on data 2010 are illustrated in Figure 3a,b. As shown in Figure 3a, each year, height increases 4 to 7 cm between the ages of 6 and 14 years. And then, the height increases 1 to 2 cm in boys aged from 14 to 17 years. At age 10 years, the values of height, arm length, chest girth, waist girth, and hip girth were, respectively, 141.4, 47.6, 72.0, 64.9, and 75.2 cm with almost 80% growth rate percentage.

As shown in Figure 3b, at age 6, height reached approximately 70% of the adult height size, and chest girth, waist girth, and hip girth reached, respectively, 67.2%, 72.2%, and 67.4% of the adult size (adults of ages 17 to 18 years). On average, maturity in growth was reached at the ages of 17 to 18 years for males.

### The size comparison between 2010 and 2013 data

**Figure 4:**
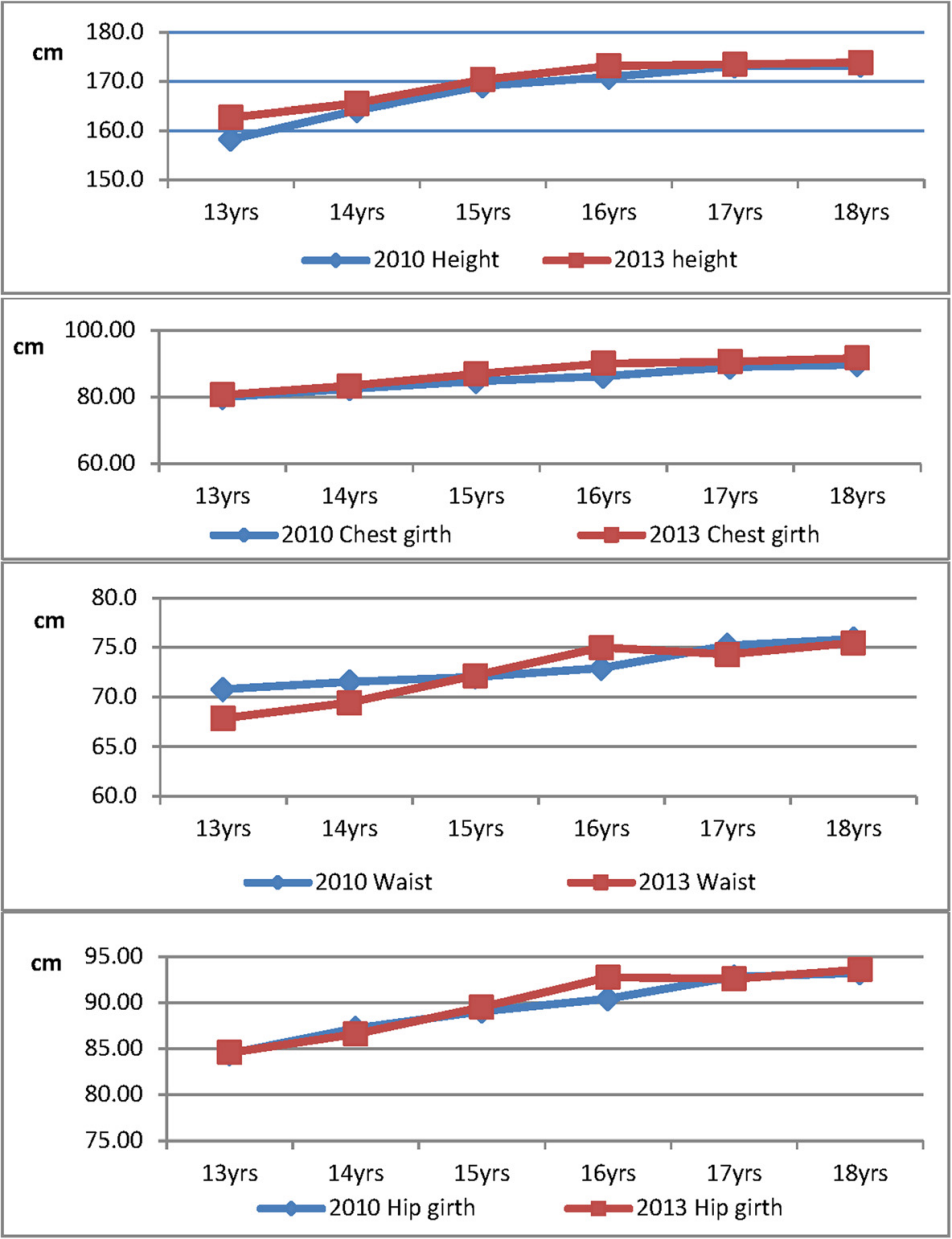
Comparison of key dimensions between 2010 and 2013 data of Korean male plotted against age.

The comparison of the mean values of height, chest girth, waist girth, and hip girth measurements between 2010 and 2013 data, which present body development features, and annual growth changes of measurements by ages 13 to 18 years in Koreans, are shown in Figure 4.

According to the 2010 data, the average chest, waist, and hip girth of 13-year-old boys were 80, 71, and 85 cm, respectively. In 2013, the sizes of 13-year-old subjects were 80.5, 67.1, and 84.5 cm, respectively.

As for the data of 16 years, the average height, chest, waist, and hip girth from 2010 data were 170.8, 86.2, 72.9, and 90.4 cm, respectively. In 2013, the results for the 16 years were 173.1, 90.0, 74.3, and 92.7 cm, respectively. In comparison with the 2010 data, which showed an average stature of 170.8 cm, there has been a 2-cm increase in height. For the girth measurements, as shown in Figure 4, compared to the 2010 data, the mean chest girth of the 2013 data at ages 16 and 18 years increased about 4 to 2 cm.

**Figure 5:**
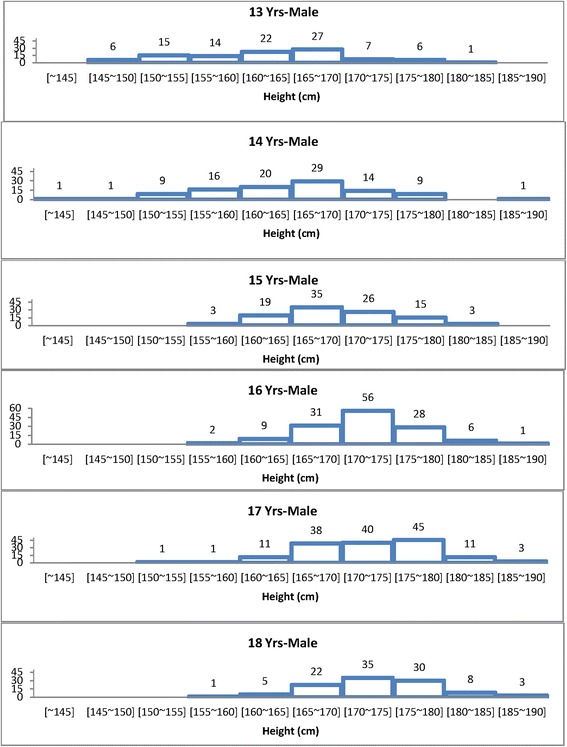
Comparison of height distribution from different age groups.

The distribution range with a 5-cm interval of body height in subjects from 13 to 18 years (data 2013) is illustrated in Figure 5. The height value range of 13-year-old males is distributed with the range of 27% from 165 to 170 cm and with a 22% cover range from 160 to 165 cm height. The height value range of 14-year-old males is distributed with the range of 29% between the heights 165 and 170 cm and with a range of 20% between the heights 160 and 165 cm. The height value range of 15-year-old males is distributed with the range of 35% between the heights 165 and 170 cm and with a range of 26% between the heights 170 and 175 cm. The height value range of 16-year-old males is distributed with the range of 23% between the heights 165 and 170 cm and with a range of 42% and 21% from the heights 170 to 175 cm and from 175 to 180 cm, respectively. At 17 to 18 years, the height is attained to that of the adult size with a 35% range of 170 to 175 cm and a 30% distribution of 175 to 180 cm.

Data are divided into three zones corresponding to different height types of Korean 13- to 18-year-old males. The three body shape ranges in descriptive titles are as follows: from R (regular), T (tall), and S (small). Type R, whose height value ranges from 165 to 170 cm, can be defined as the regular size for 13- to 15-year-old males, and whose height value ranges from 170 to 175 cm, can be defined as the regular size for 16- to 18-year-old males. Type T, whose height value ranges greater than 175 cm, can be defined as the tall type for Korean males aged 16 to 18 years. Type S, whose height value ranges smaller than 165 cm, can be defined as the small size for 13- to 15-year-old males, and whose height value ranges smaller than 170 cm, can be defined as the small size for 16- to 18-year-old males.

### 3D body silhouette modeling from 2013 data aged from 13 to 18 years

**Figure 6:**
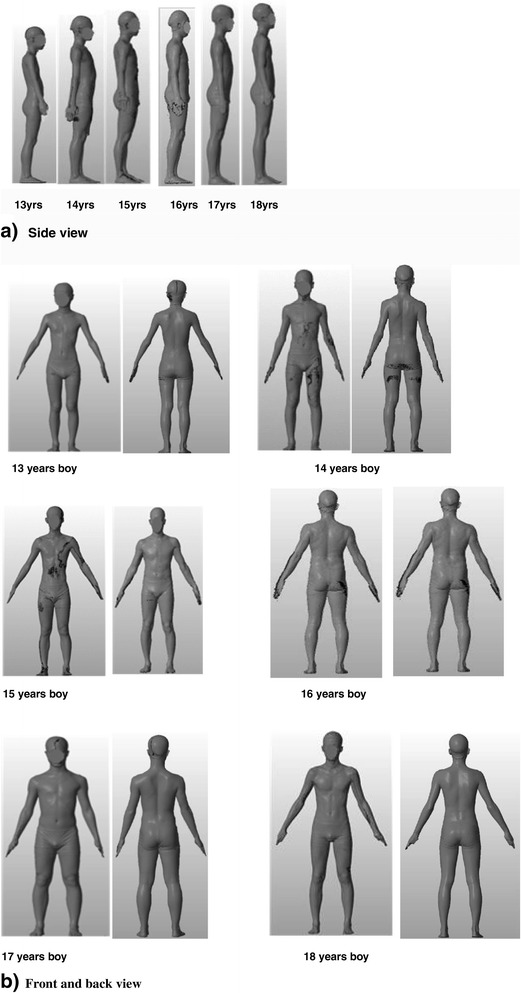
Body silhouettes modeling at different ages. **(a)** Side view. **(b)** Front and back view.

Figure 6 shows an example of 3D body shape modeling of Korean in their 13 to 18 years. As shown in Figure 6 and Table 1, the means of height, chest, waist, and hip girth are, respectively, 173.2, 91, 77, and 95 cm in boys aged 17 years, and, respectively, 166, 84, 72, and 90 cm in boys aged 14 years. The drop values calculated the differences between chest girth and waist girth showed 12 cm aged from 13 to 14 years, 13 cm at age 15 years, 14 cm aged from 16 to 17 years, and 15 cm at age 18 years. These sizes and body shape can be regarded as the 50th percentile young adult body shape in Korean as shown (photos from ‘Size Korea 2013’).

**Table 1:** **Body size of 13- to 18-year-old boys (from 2013 data)**

	**Body size(cm)**					
	**Age (years)**	**Height**	**Chest**	**Waist**	**Hip**	**Chest-waist (drop value)**
Male	13	163	80	68	87	12
	14	166	84	72	90	12
	15	169	86	73	92	13
	16	171	90	76	94	14
	17	173	91	77	95	14
	18	173	92	77	94	15

### Body height proportions corresponding to total height

**Figure 7:**
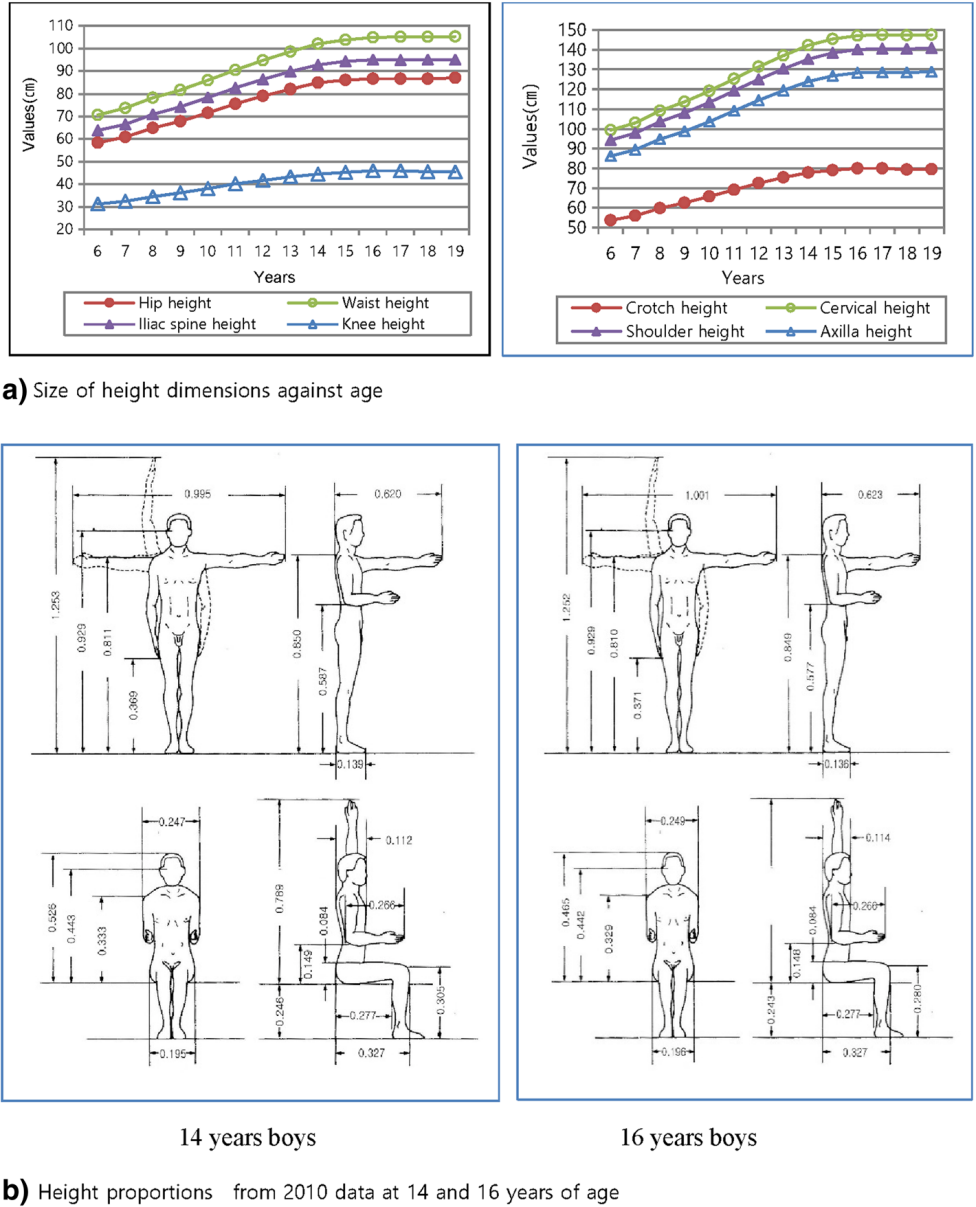
Size of height dimensions **(a)** and body proportions **(b)**.

Figure 7a shows the size changes of height dimension in shoulder height, waist height, crotch height, cervical height, knee height, axilla height, hip height, and illiac spine height based on 2010 Korean male data set. The values of waist height, shoulder height, crotch height, cervical height, knee height, axilla height, illiac spine height, and hip height of the male group in their 17 to 18 years were 105, 140.4, 79.9, 147.5, 45.7, 128.4, 94.8, and 86.5 cm, respectively.

Body proportion is critical in the manufacture of body-fitting clothes. The proportion ratio index of body height dimension corresponding to total body height should be taken into consideration when designing good product construction systems. Five body dimensions, namely, total body height, eye height, shoulder height, fingertip height, and span and maximum shoulder breadth, are considered to be very important parameters for well-fitted balanced design and functionality. As shown in Figure 7b, the results of the 16-year-old subjects showed 0.93, 0.81, 0.38, 0.99, and 0.26 times the height in eye height, shoulder height, fingertip height, and span and maximum shoulder breadth, respectively.

### BMI

**Figure 8:**
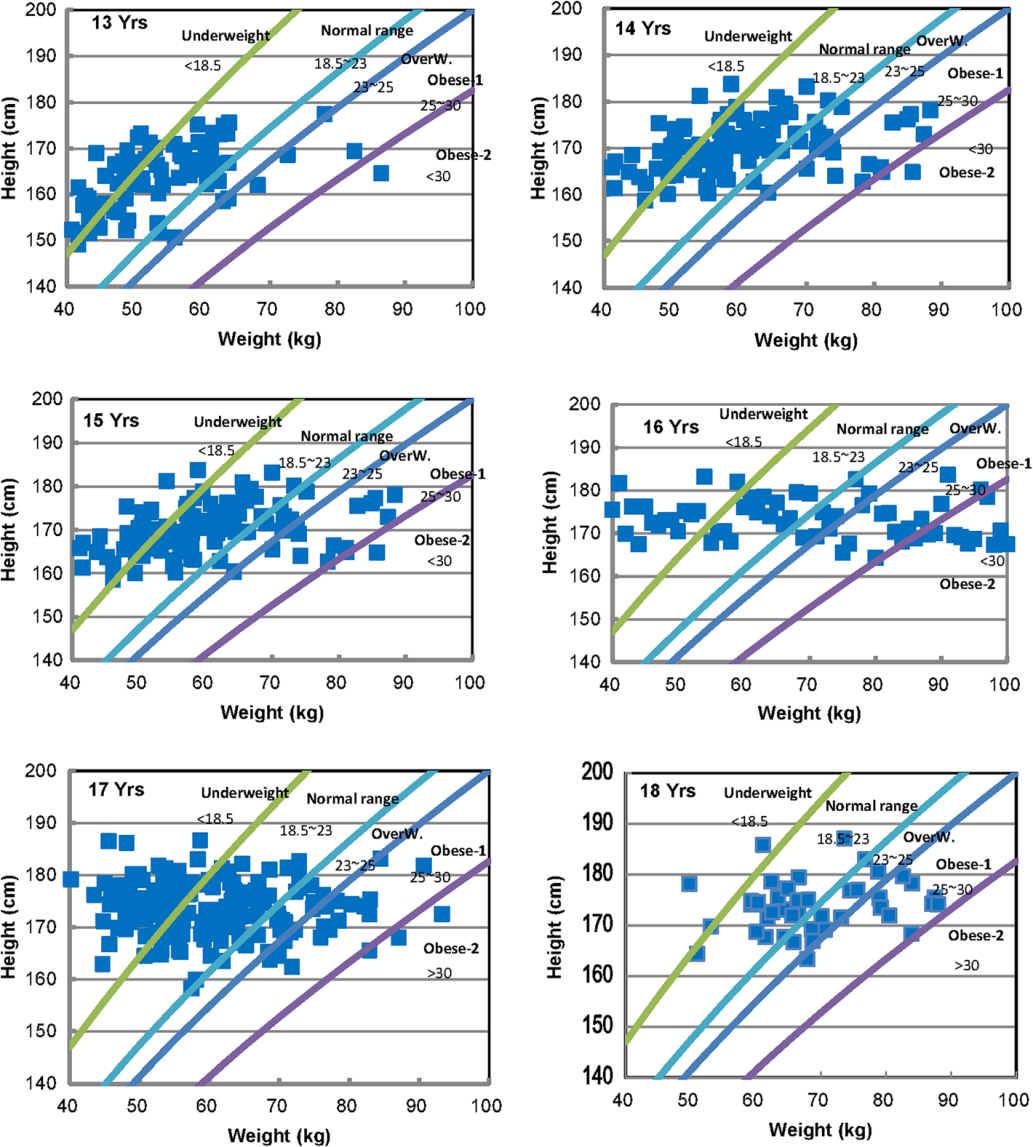
Classification of underweight, overweight, and obesity according to BMI.

The range from underweight to obese is plotted in Figure 8 and Table 2. As shown in Figure 8, the ratios of overweight subjects from ages 16 to 18 are within 15% and 17%. These ratios being roughly twice the ratios (5% to 8%) of the 13- to 15-year-old boys show thus an important increase from that age.

**Table 2:** **Classification of underweight, overweight, and obesity according to BMI**

			**Body mass index (kg/m** ^**2**^ **)**			
	**Age (years)**	**Total ** **(** ***N*** **)**	**Underweight ** **<18.5**	**Normal range 18.5 to 22.9**	**Overweight 23.0 to 24.9**	**Obesity ** **≥25**
Male	13	97	37(38%)	50(52%)	5(5%)	5(5%)
	14	108	35(32%)	56(52%)	8(7%)	9(8%)
	15	111	25(23%)	63(57%)	9(8%)	14(13%)
	16	159	23(14%)	80(50%)	24(15%)	32(20%)
	17	199	22(11%)	111(56%)	34(17%)	32(16%)
	18	114	14(12%)	58(51%)	17(15%)	25(22%)

Adopt the classification from WHO.

## Discussion

Good anthropometric surveys are difficult to conduct, expensive, and, when applied to civilian population, few in number. Large samples have been taken from the armed forces to create man/machine relationships successful in a fitting environment. Although these measurements have been extensively taken, they are limited to the selected groups, for example, pilots. Civilian surveys are not extensive in terms of samples and measurements, and most are out of date. Regarding military populations, surveys have been carried out on military personnel of France [20], Germany [21], and of the UK, particularly by the RAF [22].

In the Far-East, surveys have been conducted on military personnel of Korea, Japan, Thailand, Vietnam, and India, and in the Middle East, on Iranian army personnel. As regards civilian populations, Australia has been fairly well covered [23].

Nowadays, the extensive surveys have been carried out on the principal population groups (that is, men, women, and children) using the 1D and 3D method as shown in Table 3.

**Table 3:** **Size surveys from countries**

	**UK**	**USA**		**Japan**			**Korea**	
Survey year	2001 to 2002	2002 to 2003	2007 to 2008	2004 to 2006	2010	2011	2003 to 2004	2010 to 2013
Organization	3DEC Center	TC2	NHNES	HQL			KATS	KATS
Samples (*N*)	11,000	12,000	9,700	6,700	109	149	5,168	4,400
Age range (years)	Over 16 years adults	Adults	All ages	18 to 90	20 to 58	Foreigners living in Japan	8 to 75	7 to 69
Methods	−1D	−3D	−1D	−1D	−1D		−1D	−1D
	−3D			−3D			−3D	−3D

From these results, comprehensive tables have been compiled showing the percentage of population distribution for particular sets of body dimensions and anthropometric features for the comparison of the size information between countries and for a best fit and function for products.

It would be interesting to know if the size survey information of each country was similarly controlled by the size factors and had equal growth tendency similarity. As there is little published data on this, we have carried out an analysis of variance on height and three key girth dimensions in the growing children’s Korean population samples.

In Korea, like many other studies [24,25], the mean height is concerned of primary importance for all growing children to define the features of growth. As can be seen in this paper, the mean height increased about 6 cm from 12 to 14 years which shows the early fast maturing somatotype compare to the 1997 data [13]. And then, the growth tempo of mean height of boys aged from 15 to 17 years increased 1 to 2 cm to a level slightly down during this year. Growing of the body reached their pubertal spurts nearly a year earlier in 2010 than in 2003 [14] data.

The growth changes from ages 6 to 20 years, boy subjects are shown to grow upwards rapidly, during the height range of 135 to 140 cm (approximately 9 years). For during the years represented by the height range of 135 to 140 cm, boy subjects grow upward, rapidly remaining slim, while in the latter years, they tend to consolidate or broaden. As plotted in Figure 3, height increases from 6 years to maturity 1.5 times mainly due to rapid growth in leg length. On average, maturity of growth in height was reached at the ages of 17 to 18 years for males.

Tanner and Hayashi [26] showed the good estimation of the age at maximal pubertal increment of the population as well as of final height using the mean heights of school children measured in 1957, 1967, and 1977.

Tanner and James [27] indicated that in the interpreting differences in height between different populations of children, a fundamental distinction has to be made between growth tempo and size itself. Some children run through the whole period of growth rapid tempo, others a slower one. The rapidly growing children reach puberty at an early age and cease growing soon; slowly growing reaches puberty later and keep on growing for longer. The important thing to note is that among normal, healthy children, tempo and final height are totally independent.

In Korean boys, increasing annual growth in chest, waist, and hip girth, with 3.80, 3.11, and 3.70 cm, respectively, at ages 9 to 10 (with height of 135 to 140 cm) corresponds to male child-adult transformation in body shape. The body girth dimensions exhibit a jump in magnitude at the 140 cm height primarily for chest and hip development combined with the normal body ‘consolidation’ that occurs in boys. There have been increases in growth increment in the more recent period compared to 1986 data [11]. And there is a definite trend toward earlier maturation and greater total body build. A number of studies have been carried out on the growth stages of puberty [28-30]. These reports showed that the puberty stages for boys appear at ages 9.1 to 12.5 years, while girls reach the stages as early as 10.0 years. The growth stages are highly correlated to height growth as shown in Figure 4. Total body height is clearly of primary importance for all growing children for clothes-fitting purposes, and this has been recognized in many national standards [31,32].

The three body height ranges in Korean boys with descriptive titles from R (regular), T (tall), and S (small) shown in Figure 5 and the 50th percentile of Korean youth with different body silhouettes shown in Figure 6 are as large as Europeans in height [33].

As shown in the results (Figure 7), small differences in body proportions at birth are continuously multiplied by differential growth tempo up until maturity [29], after which body shape changes are influenced by age, quality and quantity of food intake, exercise, and social conditions. It is to be expected that limb development progresses proportionately with height development or approximately so.

For the results of height proportions against to the total height, the values of 16-year-old subjects showed 0.93, 0.81, 0.38, 0.99, and 0.26 times the height in eye height, shoulder height, fingertip height, and span and maximum shoulder breadth, respectively. These values can be used to make work space and accessory designs in order to predict the body length of each part [34].

Figure 8 shows the body mass index of 13 to 18 years of age. The ratio of the overweight group is increased from the age of 16 years. This reality may be reflective of societal influences because targeting obesity in both children and adults has become a major public health focus over several years. This pattern is consistent with recent Korean cultural emphasis on health and physical fitness according to the economic development. The causes of the observed trends, insofar as they have been identified, are related to cultural processes.

In other studies [35,36] on body size and BMI of children, Ambrosi suggested that the correlation between perception of personal body size and corresponding BMI was significant for boys. Therefore, more research between body shape and BMI is needed to give perceptions related to obesity for young generations.

## Conclusion

This paper dealt with growth pattern of height, key dimension distributions according to height, and morphological growth patterns of 6- to 20-year-old Korean boys using the KATS data taken from 2010 survey. We also investigated the comparison of the BMI, body silhouette images, and the difference of mean heights between 2010 data and 2013 data of 13- to 18-year-old boys based on the anthropometric data samples of KATS taken from the 2013 survey.

As indicated in another research, because individuals vary so much in the age at which they reach adolescence and because adolescence involves such relatively large changes in body size, physiological function, and social behavior, morphological age as applied to size is a rather obvious concept. A ‘height developmental age’ can be easily obtained by finding the age at which the given child’s actual stature equals the height of the average child, the measure has limited usefulness, as it confounds maturity with size. The concept of ‘shape age’ is a more subtle and more rewarding one. We have to distinguish differences in proportion due to growing from differences in proportion that distinguish adults. But the changes of finding growth changes independent of adult differences seem greater with shape than with size [29]. The results of this paper show the same tendency with this analysis.

There are also several studies which cover growth features of the entire range from birth to maturity, and they have reported the growth at adolescence and the comparison of the growth patterns among European. Even though such researches have been made, nowadays, the human modeling tools based on the anthropometric data and morphological features that cover all the countries should be developed for industrial purposes, for well-fitting garments and other human-oriented design process.

## Abbreviation

KATS: Korean Agency for Technology and Standard
